# Fostering Tobacco Regulatory Team Science through a multisite, virtual fellowship program for early-career researchers

**DOI:** 10.1017/cts.2021.887

**Published:** 2022-01-04

**Authors:** Tinuola B. Ajayi, Ellen Childs, Christy Di Frances Remein, Leigh R. Forbush, Justin B. Ragasa, Jessica L. Fetterman, Glenn A. Hirsch, Emelia J. Benjamin

**Affiliations:** 1 Boston University School of Medicine, Boston, MA, USA; 2 Boston Medical Center, Boston, MA, USA; 3 Abt Associates, Rockville, MD, USA; 4 George Washington University School of Medicine and Health Sciences, Washington, DC, USA; 5 Seed Global Health, Boston, MA, USA; 6 National Jewish Health, Denver, CO, USA; 7 Boston University School of Public Health, Boston, MA, USA

**Keywords:** Tobacco Regulatory Science, American Heart Association, mentoring, curriculum, multi-institutional

## Abstract

**Introduction:**

In an era of complex, multi-institutional, team-based science, there is little guidance for the successful creation of effective, collaborative, multisite training programs.

**Objective:**

We designed, implemented, and evaluated a multi-institutional Tobacco Regulatory Science (TRS) fellowship representing a scalable program that may be customized for other research areas.

**Methods:**

Using a mixed-methods approach, we analyzed program evaluations from trainees enrolled in the first 7 years of the American Heart Association (AHA) Tobacco Regulation and Addiction Center (A-TRAC) fellowship (2014–2021). We also reported the program outcomes, including published TRS manuscripts, independent grant funding, Food and Drug Administration (FDA) Docket comments submitted on TRS topics, TRS oral and poster presentations, research awards, and promotions in the TRS field.

**Results:**

Thirty-five unique trainees (49% [n = 17] female, 29% [n = 10] Black) from eight institutions within the A-TRAC network participated in the fellowship since its inception. The trainees reported 74 TRS publications, 78 TRS oral or poster presentations, 25 FDA Docket comment submissions, and 13 funded grant awards. Participant evaluations indicated six areas of programmatic strength: 1) blended instruction medium with webinars and in-person meetings, 2) curricular emphasis on theories of experiential learning, 3) focus on career and professional development, 4) integrated mentorship model, 5) culture of feedback and feedforward to foster successful learning, and 6) focus on recruiting diverse participants. The A-TRAC model stresses experiential education, feedback and feedforward, and peer learning.

**Conclusions:**

Our resource-effective, needs-driven program is a reproducible model for institutions interested in developing multisite, virtual research education programs in the era of team science.

## Introduction

With the emergence of new tobacco products in the market over the last two decades, Tobacco Regulatory Science (TRS) has become a vital discipline of fundamental importance to public health [1]. In order to reduce the public health burden of tobacco in the USA, the Food and Drug Administration (FDA) has committed to support science and research to further understand tobacco use and associated risks [2]. In 2013, the FDA’s Center for Tobacco Products and the National Institutes of Health awarded several institutions research grants to produce the evidence base required for tobacco product regulation to protect public health [3]. The interagency collaboration launched the Tobacco Centers of Regulatory Science (TCORS); 14 centers in 2013 (TCORS 1.0) and nine centers in 2018 (TCORS 2.0) were funded to provide scientific evidence for tobacco product regulation [4,5]. The initiative created an urgent need for scientists with the skills to generate multidisciplinary tobacco research to inform regulatory policy.

In an effort to successfully create an effective, collaborative, educational program, in 2014 we developed the American Heart Association (AHA) Tobacco Regulation and Addiction Center (A-TRAC) fellowship, a multisite, virtual program to train early-career biomedical and population researchers working across complex, interdisciplinary areas of TRS. We implemented a blended education model involving weekly webinars and annual in-person meetings, with a focus on experiential learning techniques, mentor network development, and the value of feedback for scientific excellence and career advancement.

Given the broad educational requirements of TRS training and the geographically diffused network of TRS researchers [6], the A-TRAC program was designed to capitalize on developments in online education. Increased innovations in interactive online educational tools have dramatically affected all areas of education, including biomedical research education [7]. Although the need for trained medical professionals is universal, research education programs often are concentrated within large cities, making online program delivery a helpful application in research education and training to less populated regions [8]. Through web conferencing technology, the provision of early-career research and medical mentorship from experienced health professionals has become possible, irrespective of geographic location [9,10]. The current COVID-19 pandemic and the resulting lockdown mandates have accelerated dramatically the need for online learning and evidence of efficacy at every level in the education spectrum [11]. By engaging in our virtual fellowship program, early-career TRS scholars are provided with a forum to regularly meet and engage with national leaders in the field.

**Fig. 1.**
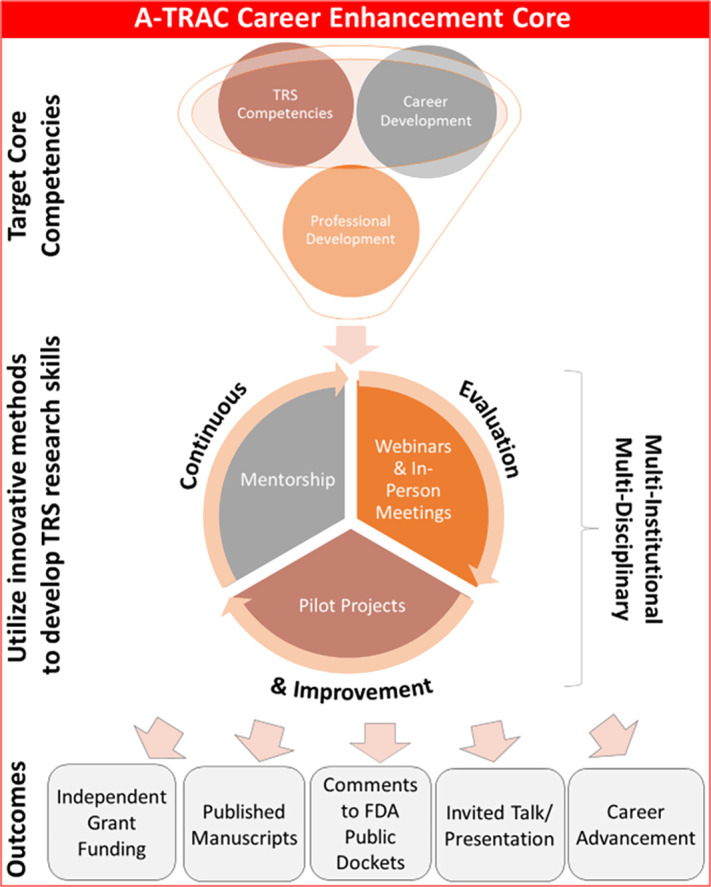
A-TRAC fellowship core competencies. A-TRAC, American Heart Association Tobacco Regulation and Addiction Center; FDA, Food and Drug Administration.

The A-TRAC training program comprises three primary elements: 1) TRS core competencies [6]; 2) career development related to competencies essential to pursuing a scientific career, such as conducting research, abstract/manuscript/grant writing, scientific presentations, and submitting FDA Docket comments (remarks on drafts for proposed FDA regulation and rules before they are promulgated) [12] ; and 3) professional development skills, such as developing robust mentoring networks, time management, difficult conversations, and giving effective feedback and feedforward (Fig. 1). Because TRS is a relatively new field, TCORS has defined TRS competencies to guide and align education programs, including topics related to tobacco control, health, addiction, marketing, and litigation [6]. Additionally, preliminary research suggested that a TRS-specific mentorship training program has the potential to fulfill heterogeneous educational requirements [13].

Using systematically collected program evaluation data from the first 7 years of the A-TRAC fellowship (2014–2021), we describe the program’s components and outcomes. Conceptually, the A-TRAC model need not be discipline specific; rather, it functions as a customizable archetype, providing translatable guidance for developing virtual fellowship programs that seek to combine specialized content knowledge, career and professional development, and intensive mentorship in a highly resource-effective manner.

## Methods

### A-TRAC Multi-Institutional Structure

The Career Enhancement Core (CEC) is the training core within the A-TRAC grant, charged with leading the fellowship. It is the faculty team responsible for coordinating the fellowship’s educational activities. The CEC is comprised of A-TRAC investigators and administrative staff. For both iterations of the grant, A-TRAC 1.0 [2013–2018] and A-TRAC 2.0 [2018–2023], the CEC has been tasked with developing the next generation of basic, clinical, population, and translational investigators in tobacco-related cardiovascular health and policy and to accelerate TRS. Trainees gain expertise in both scientific investigation and public health to inform the FDA Center for Tobacco Products with relevant findings for regulation of the manufacture, distribution, and marketing of tobacco products. We leveraged the outstanding expertise, facilities, infrastructure, and research experience at participating institutions within the A-TRAC grant (Boston University, Wake Forest University, Johns Hopkins University, New York University, University of Louisville, Tougaloo College, Florida International University, and the University of Mississippi Medical Center) to build a robust training curriculum. We also utilized the broad portfolio of these AHA-affiliated institutions’ research and training programs by providing opportunities in networking, research collaborations, and peer mentoring for the A-TRAC fellowship trainees.

In addition, the CEC focused on research of the adverse consequences of cardiovascular disease from diverse tobacco products/uses. We worked with other TCORS sites to develop a rigorous, innovative multi-institutional career and research development program for trainees.

### Recruitment of Trainees

The CEC accepted applications including project proposals for funded and alternatively funded candidates. Some candidates were funded directly through the grant in A-TRAC 1.0 or through CEC pilot awards in A-TRAC 2.0, whereas the alternatively funded candidates received independent funding from principal investigators within the A-TRAC grant. Both sets of trainees had the same expectations for participation and productivity. Successful applicant proposals were within the scope of A-TRAC 1.0 and 2.0 research goals and responsive to one or more of the FDA Center for Tobacco Products research priority areas [14,15].

Applications were evaluated based on the: 1) proposed project’s significance and innovation; 2) candidate’s demonstrated ability to carry out the proposed project; 3) applicability of research to A-TRAC’s mission and scientific scope; 4) compliance with FDA’s regulatory authority and alignment with FDA Center for Tobacco Products research priority areas; 5) strength of research and mentoring teams; and 6) inclusion of a clear and realistic timeline for investigating and publishing the project. All A-TRAC trainee pilot projects required FDA Center for Tobacco Products and NIH approval prior to project funding.

In addition to pursuing a research project, A-TRAC trainees agreed to participate in the fellowship curriculum to learn about the scientific evidence around tobacco addiction, toxicity, health consequences, product diversity, product marketing, legislation, and economics. Institutional mentors of accepted trainees signed a mentoring commitment statement with their trainees. In addition, the trainees committed to attending 75% of the 90-minute weekly webinars and at least two in-person (virtual during the pandemic) annual meetings per year, one being the A-TRAC Annual Meeting with federal partners.

Eligible applicants included current MPH, MD, or PHD candidates, post-doctoral fellows (PhD, MD, MPH, or MSc), early-career faculty, defined as investigators with less than 5 years’ experience in TRS. The applications received were from a wide array of disciplines, including toxicology, pharmacology, public health, nursing, medicine, and dentistry. The CEC required applicants’ institutional mentors to be A-TRAC grant principal investigators [16] working at the same institution as the trainee.

### Training Model and Curriculum

Wipfli et al. defined TRS competencies across six core domains and five specialized domains to guide emerging TRS curricula and training programs [6]. Using these defined competencies, we constructed an intensive 2-year curriculum for TRS early-career investigators. The curriculum highlighted learning goals in health consequences of tobacco use and population health impact; Tobacco Control Act/FDA regulatory framework; vulnerable populations; tobacco and nicotine product diversity; and tobacco control policies and programs [6]. It also included elements of tobacco addiction, toxicology, litigation disclosure, marketing/communication, and economic cost/benefit. The curriculum was designed to concurrently support trainees’ research in tobacco science and provide them with crucial tools to pursue successful TRS careers. It was constructed to: 1) develop a strong knowledge base in tobacco science and the adverse effects of tobacco use on the human body; 2) develop an understanding of the history, legislation, and current events around tobacco use and regulation; 3) apply new knowledge to their research and scientific endeavors; 4) gain the professional tools necessary to successfully navigate careers in TRS research, policy, education, and/or advocacy; 5) further build their peer and senior mentoring networks; and 6) make meaningful progress on their proposed research projects over the course of the funding cycle.

A needs assessment performed through an entrance survey at the beginning of each academic year also provided an opportunity to further understand trainees’ needs and interests relevant to TRS professional development skills. In addition, the needs assessment informed current and future expansion of our programming. Questions in the needs assessment are listed in Supplement 1.

We utilized a blended-learning, “flipped classroom” (pre-session readings and preparation, experiential learning during webinars) approach to accelerate trainees’ career development [17] (Table 1). Participants met by video-conference weekly with investigators across a variety of academic and professional fields to develop the above-mentioned skills and competencies. These webinars utilized readings, videos, presentations, activities, and projects to maximize the trainees’ learning experience. During the last half hour of each webinar, a designated trainee updated the cohort on the progress of their research through a “work in progress” presentation which included successes and challenges, and then engaged in a feedforward [18] activity. To distinguish feedforward from feedback, the feedforward process involves reflection on internal standards of excellence based on past performance and the necessary steps the individual needs to attain these standards toward future goals. With feedback, there is an external standard the individual is being evaluated against and is given steps to attain those standards [18]. Feedback is typically focused on past behavior or performance. Feedforward emphasizes examining options for future performance and goals. The work-in-progress session is one of many opportunities for each trainee to recruit peer and senior feedback and feedforward on their project and practice presenting scientific research to diverse audiences.

**Table 1.** Examples of TRS curriculum: topics, objectives, and related experiential activities for weekly webinars

Topic	Learning objectives	Experiential activity
Dockets	Describe the process for submitting a comment to the FDA Dockets Write and submit a comment to the FDA Dockets	Select an FDA Docket currently open for public comment. https://www.regulations.gov/ Read a section of the proposed policy and write a draft comment (<1 page) on the scientific evidence that supports/refutes the proposed policy. Send to Director of Training and Education the morning before the webinar. Workshop these Docket comments and give feedback during the webinar Participants then revise and submit the Docket comment after the webinar
Data visualization	Determine what data and statistical results are best displayed in text, tables, and figures Identify design features of effective tables and figures Create tables and figures to summarize statistical results Practice improving data presentation for both scientific and lay audiences	Prepare the same data in two different figures – one for a lay audience and one for a scientific audience Send to Director of Training and Education the morning before the webinar. Workshop these figures and give feedback during the webinar
Tobacco and cardiovascular principles	Identify the health consequences of active and passive smoking	Complete assigned pre-webinar resource assignments and come prepared to discuss the following: What might the FDA look for in terms of biomarkers or subclinical measures of atherosclerosis or toxicity from smoking? What evidence would the FDA need to form policies using biomarker data?
Marketing – translating basic science for the clinical realm and to individuals who smoke	Learn how to make TRS research and science accessible to patients, policy makers, and the general public Apply marketing principles to personal research and TRS work	Ahead of webinar, in small, assigned groups: Pick one of the seven presented advertising techniques and a target audience to build an effective anti-smoking ad Develop a Strategy Brief to provide background and direction for advertising execution, utilizing consumer insights Share and workshop the ads during the webinar
Grant writing	Develop strategies to write effective proposals for funding	Create a draft Specific Aims to workshop during the webinar. Identify 1 strength and 1 area for improvement for the Specific Aims page Comment on how effectively the Specific Aims address the hypothesis/overall goals

A-TRAC, American Heart Association Tobacco Regulation and Addiction Center; FDA, Food and Drug Administration; TRS, Tobacco Regulatory Science.

The A-TRAC program elements align with the TRS training support provided by Center for coordination of Analytics, Science, Enhancement, and Logistics (CASEL) in TRS. The CASEL TRS Knowledge Center is a centralized online resource for over 1000 TRS investigators where they can access TRS-related events, announcements, learning materials, job postings, funding opportunities, data, and measure resources [19]. CASEL provides additional training content spanning the TRS competencies but lacks the experiential component implemented by the A-TRAC program. For trainees who are completing academic degrees alongside the fellowship, the A-TRAC experience proves complementary by providing them with broader educational input from national thought-leaders in the field, as well as with a more expansive peer and senior mentoring network to supplement any programmatic mentoring they already receive. When designing the scholastic activities associated with our curriculum, we do not operate on the assumption that trainees already have Individual Development Plans (IDPs); hence, they design IDPs at the beginning of each academic year. Their IDP serves as a guide for their long- and short-term goals for the duration of fellowship.

A recurrent feedback theme from the trainees over the last few years was the desire to closely collaborate with their peers across institutions. In 2020–2021 academic year, we piloted “collaboration pairs” among the cohort of trainees with the permission of their institutional mentors. The concept involved having an assigned partner from another institution who would be included as collaborator and potential middle author on a trainee’s project. Other ideas for collaboration included manuscript/grant writing and review.

Another essential aspect of the curriculum is the face-to-face meetings during the academic year. Trainees committed to attend at least two in-person/virtual annual meetings over the course of the fellowship to enhance their ongoing learning and increase their exposure to professional efforts or networks in tobacco science. Pre-pandemic in-person/virtual meetings occurred during the AHA Scientific Sessions, Fall TCORS Grantee Meeting, and A-TRAC Annual Meeting.

### Multilevel Mentoring Network

**Fig. 2.**
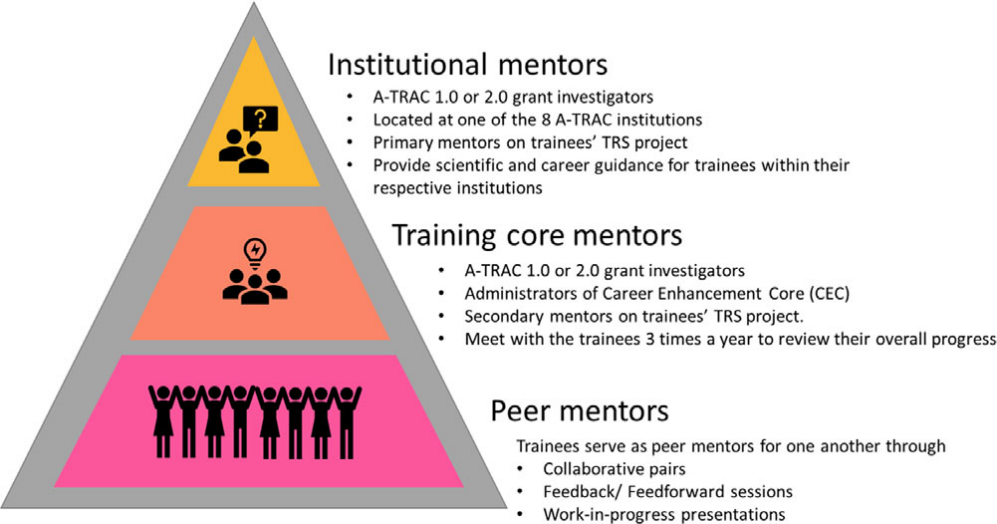
Multilevel mentorship framework. A-TRAC, American Heart Association Tobacco Regulation and Addiction Center; TRS, Tobacco Regulatory Science; CEC, Career Enhancement Core.

Russo et al. [13] reported challenges specific to TRS mentoring, including conveying unique aspects of TRS to mentees and constructing policy-responsive research questions and proposals. In response, we developed TRS mentor training modules for investigators engaged in mentoring TRS early-career researchers using outlined TRS competencies [20]. We also integrated a multilevel mentoring framework into the curriculum. The mentoring network was comprised of training core mentors, institutional mentors (scientific and career), and peer mentors within each cohort (Fig. 2). This unique framework focused on regular, structured mentoring sessions to improve research skills and achieve professional goals, including national presentations, peer-reviewed publications, submission of comments to FDA Dockets, and grant submissions. A critical level of mentorship was via the institutional mentors. Before being formally admitted to the program, trainees had to meet with their institutional mentors to review and sign mentoring agreements. In conjunction with trainees’ personal commitment statements, mentoring agreements represented a commitment to investing in the program to reap the greatest possible educational benefits.

The CEC mentors served as another level of mentorship. Our program provided biannual check-ins among mentors, mentees, and CEC personnel to scrutinize the strengths and opportunities to improve the mentoring relationship, the mentee’s productivity, and opportunities to improve the fellowship. Additionally, trainees served as peer mentors, providing accountability and feedback on the mentee’s progress, opportunities, and challenges.

### Evaluation

To evaluate the impact of our approach, we used mixed methods to conduct a robust educational and program evaluation. We employed the evaluation to promote continual improvement and innovation in TRS training while collaborating with the cross-TCORS training workgroups. Data from evaluations were analyzed and used in real time to facilitate ongoing CEC curriculum and programming quality improvement. The evaluation was designed to assess the curriculum’s efficacy, which was constructed based on TRS competencies. By evaluating the individual sessions, we indirectly evaluated the broader TRS competencies defined by Wipfli et al. [6].

As part of the ongoing A-TRAC program evaluation, the training coordinator systematically distributed and collected evaluation materials. We conducted: 1) entrance surveys and needs assessments for new trainees; 2) weekly post-webinar evaluations to evaluate the effectiveness and value of weekly webinars; 3) mid-point and end-of-program satisfaction surveys to measure satisfaction related to overall CEC programming; 4) biannual trainee progress reports completed collaboratively by mentors and mentees to assess the progress of trainees; and 5) mid-point and end-of-program mentoring web-based check-ins between A-TRAC CEC mentors and trainees to assess the effectiveness of our mentorship structure.

Trainees’ progress was assessed based on their stated goals, as well as in the following areas: published manuscripts, independent grant funding, FDA Docket comments submitted on TRS topics, TRS oral and poster presentations at conferences or educational venues, and research awards, recognition, or promotions in TRS field. Evaluations from 2014 through 2021 were reviewed and analyzed for relevant themes.

### Data Analysis

We analyzed all qualitative evaluation materials using NVivo qualitative data analysis software (QSR International Pty Ltd Version 10, 2012). Using a grounded theory approach [21], one study team member reviewed and coded all documents to develop an initial coding scheme. Using the initial coding scheme, another study team member independently coded additional documents and suggested additional relevant codes. During this process, the study team met to discuss all codes and included relevant codes about TRS competencies [6]. The two members of the study team used the coding scheme to recode all remaining documents, employing an iterative process to discuss new categories that emerged in the transcripts until they reached consensus. Quantitative metrics were measured for program outcomes using absolute counts and percentages. The study received approval from the Boston University Medical Campus Institutional Review Board, and the A-TRAC trainees gave informed consent.

## Results

From 2014 to 2021 (A-TRAC 1.0 and A-TRAC 2.0), the fellowship accepted 35 trainees from eight institutions within the A-TRAC network (Table 2). The trainees were 49% (n = 17) female and were racially diverse, including 46% (n = 16) White, 29% (n = 10) Black, and 17.1% (n = 6) Asian individuals. There was a wide range of highest degree at application, with the majority being MD-MPH (34%, n = 12) and PhD (31%, n = 11). Regarding their career stage at application, 66% (n = 23) of trainees were post-doctoral researchers, followed by faculty (14%, n = 5), research associates (11%, n = 4), and doctoral students (9%, n = 3). Trainees were involved in various types of TRS research: Population Science (63%, n = 22), Basic Science (23%, n = 8), Translational Science (11%, n = 4), and Clinical Science (3%, n = 1). The most common TRS field was Public Health (51%, n = 18).

**Table 2.** 2014–2020 demographic descriptions of A-TRAC trainees

	Characteristic	*N* = 35	Percentage
Sex	Female	17	48.6
	Male	18	51.4
Race	White	16	45.7
	Black	10	28.6
	Asian	6	17.1
	Other	3	8.6
Highest degree at time of application	Masters’/MD candidate	2	5.7
	MPH/MS/MA	4	11.4
	PhD	11	31.4
	MD	2	5.7
	BDS and MPH	1	2.9
	MD and MPH	12	34.3
	MD and PhD	3	8.6
Career stage	Research associate	4	11.4
	Post-doctoral researcher	23	65.7
	Faculty	5	14.3
	Doctoral student	3	8.6
Type of research	Basic science	8	22.9
	Translational science	4	11.4
	Population science	22	62.9
	Clinical science	1	2.9
Field	Physiology	1	2.9
	Communication	1	2.9
	Epidemiology	4	11.4
	Cardiology	3	8.6
	Public health	18	51.4
	Environmental sciences	1	2.9
	Toxicology	6	17.1
Type of institution	Research University	34	97.1
	Historically Black College	1	2.9
Total number of institutions		8	

A-TRAC, American Heart Association Tobacco Regulation and Addiction Center; FDA, Food and Drug Administration; TRS, Tobacco Regulatory Science.

**Fig. 3.**
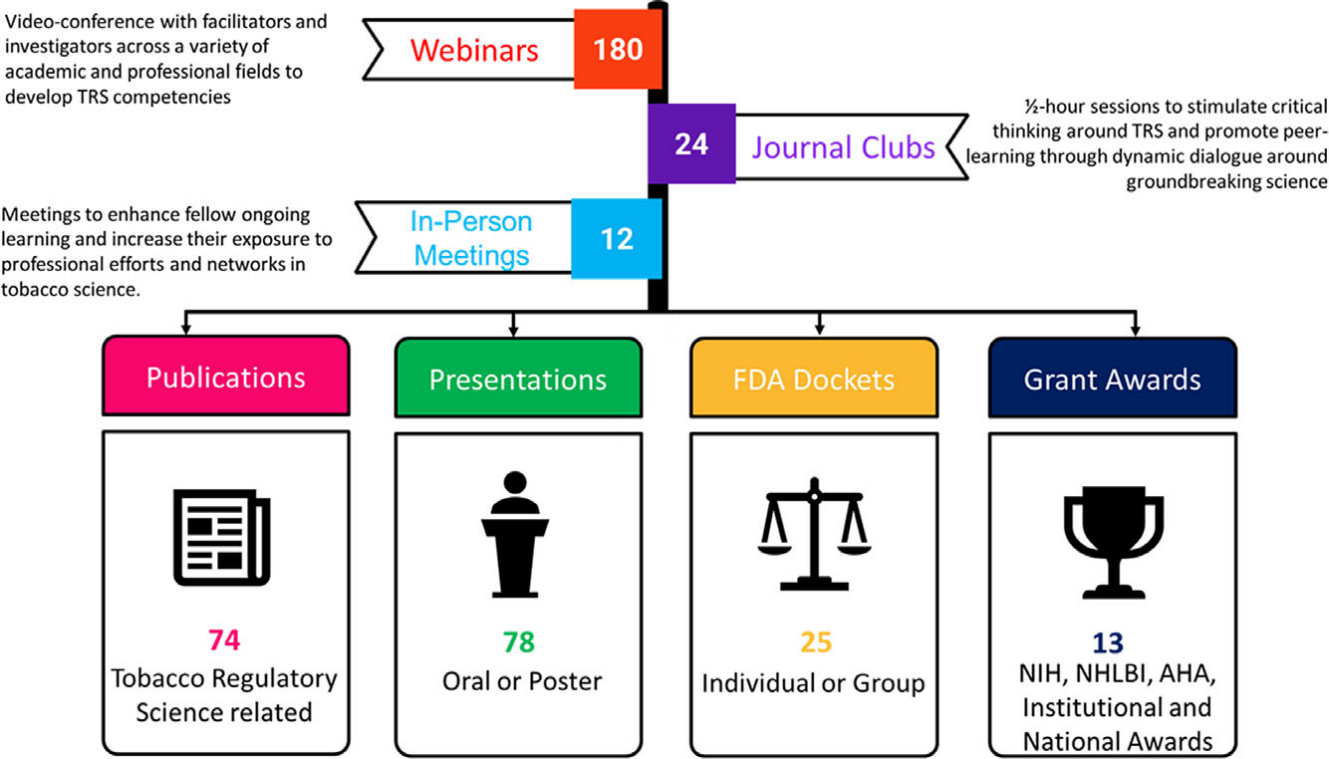
2014–2021 A-TRAC fellowship program outcomes. A-TRAC, American Heart Association Tobacco Regulation and Addiction Center; NIH, National Institute of Health; NHLBI, National Heart, Lung, and Blood Institute; AHA, American Heart Association.

**Fig. 4.**
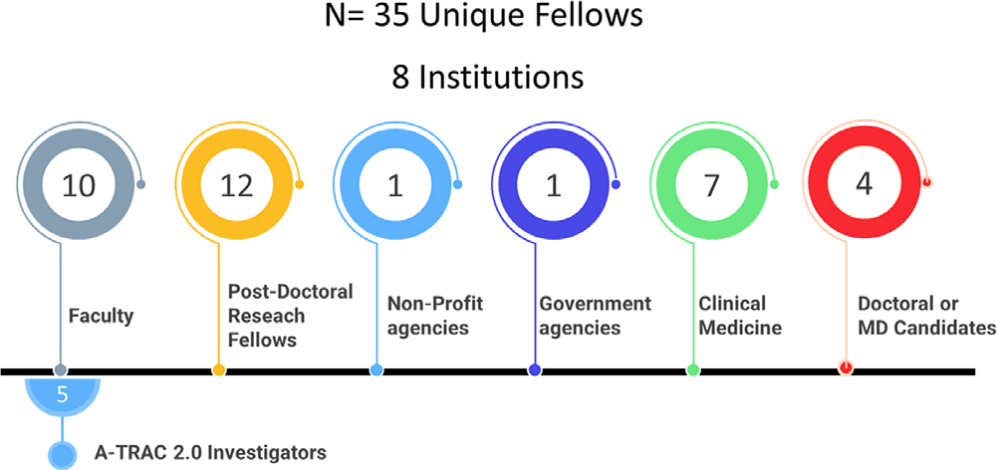
Current career of A-TRAC trainees 2014–2021 (May 2021). A-TRAC, American Heart Association Tobacco Regulation and Addiction Center.

In 7 years, we conducted 180 webinars, 24 journal clubs, and 12 in-person meetings (Fig. 3). Trainees reported 74 TRS publications, 78 TRS oral or poster TRS presentations, 25 FDA Docket comment submissions, and 13 funded grant awards (Fig. 3). As at May 2021, the current careers of A-TRAC trainees include faculty (28.6%, n = 10), post-doctoral research (34.3%, n = 12), non-profit and government agencies (5.7%, n = 2), clinical medicine (20%, n = 7), and MD and/or PhD candidates (11.4%, n = 4,) (Fig. 4). Five former trainees currently are A-TRAC 2.0 investigators.

By analyzing trainees’ program evaluations and progress reports (Table 3), we identified six key components of the fellowship: 1) a blended instruction medium, 2) experiential curriculum, 3) professional and career development, 4) intensive mentoring, 5) focus on feedback and feedforward, and 6) fellow/trainee diversity. (Supplement 2).

**Table 3.** A-TRAC trainees’ quotes related to key components of the fellowship

Component	Illustrative quote
Blended instruction medium	The weekly webinars are usually both interesting and engaging. Annual meeting and conferences were really valuable – Weekly meetings and frequent check-ins are helpful tool for accountability – Development of valuable expertise in TRS. Our in-person meetings added a personal touch to the fellowship which is often lacking in distance learning programs.
Experiential curriculum	I enjoyed how everyone said one strength and area of growth for each fellow. Then the fellow recorded it on their strip of paper, which demonstrated that your weaknesses are your strengths and vice versa when it was folded to resemble the infinity sign. I enjoyed having preparatory assignments each week that allowed us to start building knowledge and skills prior to each session. Submitting an FDA Docket comment and mentoring from [the program directors] were the most [effective] about the fellowship for me.
Professional/career development	I found the professional development and public speaking opportunities to be [effective]. Providing information and advice on how to prepare for certain steps or issues that may come across as we go through our career was very helpful, particularly for someone just starting out her career. The balance of professional development content and TRS content is a key strength of the fellowship and I enjoyed the variation in webinar topics. The fellowship is a tremendously valuable experience and has been very successful in launching my career in tobacco regulatory science. It was a transformative opportunity in many ways, including access to high-quality mentoring, scientific training, and networking.
Intensive, multi-layered mentorship	I am very much grateful and honored to be a graduate AHA fellow. I enjoyed working with and learning from my great mentors in this fantastic program. Fellowship is rigorous and on-site mentoring is [effective] The mentorship has been invaluable. Accountability has helped to achieve goals set out at the beginning of the fellowship. Having the weekly webinars, and mentoring from both [CEC faculty mentor] and my site mentor are allowing me to focus on my work, explore various avenues in tobacco research and gear towards productivity
Focus on feedback and feedforward	I enjoyed the training very much! Iʼve learned a lot and it stimulated me for personal and professional self-improvement! Thank you for your valuable time and sincere feedbacks! The guidance from the trainees and [the project PIs] has helped me tremendously. I really enjoyed the feedback from the mentors. They all had valuable insights to share on my research. The presentation sessions on the webinars help the trainees to develop presentation skills and an ability to provide constructive criticism - both useful characteristics in scientific careers.
Diversity of trainees and peer mentoring and collaboration	Meeting people from [different] institutions and opportunity to get generous feedbacks from people with [different scientific] backgrounds was particularly valuable. The [different] areas of expertise of the trainees make for an exciting and collaborative experience. The experience of moving through the program with a cohort was also very enriching and made the experience fun and stimulating. The networking opportunities at A-TRAC are endless. We as junior scientists learn more from working with other scientists than any other methods [sic] and A-TRAC fellowship provides hands on collaboration with the best ones in the field.

A-TRAC, American Heart Association Tobacco Regulation and Addiction Center; FDA, Food and Drug Administration; TRS, Tobacco Regulatory Science.

## Key Components of the A-TRAC Fellowship

### Blended Instruction Medium

A key component of the A-TRAC training core involved the hybrid of video-enabled webinars and annual in-person (virtual during the pandemic) meetings. The requirement for all participants to use webcams enhanced focus and engagement, while the blended instruction approach allowed for geographically dispersed trainees to engage in experiential activities and facilitated networking with TRS faculty across the USA. One trainee noted: “Annual meeting and conferences were really valuable – Weekly meetings and frequent check-ins are helpful tool for accountability – Development of valuable expertise in TRS.” The virtual format allowed TRS experts nationwide to participate and present on their focus areas, thus broadening trainees’ TRS networks while providing focused training from leading scholars in the field. During the COVID-19 global pandemic, the virtual format of the fellowship provided a framework for continued training with minimal disruptions.

### Experiential Curriculum

The A-TRAC training core designed the curriculum using adult-learning principles including the concepts of self-directed, project-based, and experiential learning [22]. In our analysis, a significant component highlighted by trainees was the experiential aspect. The trainees entering this program already possessed significant life experiences, and – given their diverse backgrounds – offered useful and nuanced perspectives. Because of participants’ varied expertise, emphasis was placed on methods that individualized the learning experience through innovative teaching and learning strategies. Rather than relying upon a traditional, lecture-based format, we emphasized pre-webinar background work to encourage participation in simulation exercises, case studies, problem-solving activities, and group discussion.

We worked with each module’s presenter to generate a session facilitator’s guide (Supplement 3), including learning objectives, trainees’ preparatory work, session timeline, experiential learning methods, primary post-webinar individual commitments regarding new goals and/or behavior change based on the webinar, and ongoing learning opportunities. This is exemplified in a trainee’s comment: “I enjoyed having preparatory assignments each week that allowed us to start building knowledge and skills prior to each session.” The continued learning opportunities were sometimes formulated as takeaway assignments, such as tasks to finish after the session was complete, to extend the learning process as trainees incorporated training, techniques, or strategies learned during the webinars into daily life (Table 1).

### Professional and Career Development

The A-TRAC fellowship curriculum also focused on issues of professional and career development, with themed sessions interpolated into the broader discussion of TRS competencies. Professional development webinars included topics such as leading effective journal clubs, academic writing, creating poster and oral abstracts, and grant writing/acquiring funding. More general career topics included time management, self-coaching to combat procrastination, career trajectories, elevator pitches, difficult conversations, interviewing, developing a mentor network, work-life integration, and resilience. The career advancement modules were developed using the same pedagogical methodology as the scientific sessions, with a similar focus on making sessions experiential and an even greater emphasis on gaining practical “know-how” for daily life. A trainee noted: “I found the professional development and public speaking opportunities to be [effective]. Providing information and advice on how to prepare for certain steps or issues that may come across as we go through our career[s] was very helpful, particularly for someone just starting out her career.”

### Intensive Mentorship

Given the physical distance and reliance on telecommunications for most ongoing discussions with online mentors, mentoring relationships are prone to being hampered by weak or ineffective communication, thus reducing the effectiveness of the mentorship [23]. To overcome this challenge, the A-TRAC fellowship facilitated a mentorship team, or network, to support trainees. We required all trainees to have a committed primary mentor from their own academic institution with content expertise relevant to their project. The trainees also met virtually with the A-TRAC CEC mentors annually to discuss scientific progress, roadblocks, career plans, and the individual trainee’s program goals. One trainee commented: “Having the weekly webinars, mentoring from both [CEC faculty mentor] and my site mentor are allowing me to focus on my work, explore various avenues in tobacco research and gear towards productivity.” Furthermore, webinars throughout the program included topics related to mentorship, such as expanding networks through informational interviews, developing relationships with mentors, “managing up” with supervisors and mentors, successfully navigating difficult conversations, and cultivating a range of unique mentoring relationships.

### Focus on Feedback and Feedforward

A theme raised early and often throughout the fellowship was that of the competitive advantage provided by engaging in frequent, specific, high-quality feedback and feedforward. For presentations, manuscripts, grants, and Docket comment submissions, we focused on providing *feedback* that was specific, timely, and behaviorally based. We found it useful to collect feedback from trainees through summative and formative evaluations in order to facilitate quality improvement of the programming.

In instances when trainees sought suggestions on specific challenges they were facing (e.g., recruiting participants, preparing for interviews, working with mentor), we incorporated a *feedforward* model [18,24]. Rather than focusing on past behaviors, we emphasized current and future opportunities for growth and development. A central guiding principle of professional development emphasized by the A-TRAC fellowship conceptualizes bi-directional sharing as an invaluable gift and posits that, when constructively oriented, feedforward can be a unique and powerful tool for growth.

Trainees learned how to request, receive, and give both feedback and feedforward effectively throughout experiential webinars, during peer presentations, and while meeting with their mentors. We built time into the curriculum for regular interactions regarding trainees’ work, focusing on specific comments about behavioral actions rather than on critiquing personality traits: A trainee remarked: “The presentation sessions on the webinars help the trainees to develop presentation skills and an ability to provide constructive criticism - both useful characteristics in scientific careers.”

### Fellow/Trainee Diversity

A focus on diversity and inclusion has been an important facet of the A-TRAC fellowship from the program’s inception. Based on theories grounded in the strength of interdisciplinary groups [25,26] and peer learning, trainees were selected across a wide spectrum of demographic groups, training experiences, career levels, disciplinary fields, institutional affiliations, and research areas. TRS is a broad field, encompassing diverse subject areas, from biochemistry and molecular biology to epidemiology and health policy. The A-TRAC training core fostered a community that included researchers from across a range of disciplines, providing space for participants to learn from each other through sharing their unique knowledge and expertise. Early research shows the cooperative social exchange between equal partners and more experienced peers is vital in peer learning [27–30]. A-TRAC trainees varied by career stage, ranging from MPH students to early-career MD/PhD faculty members.

Participants also were geographically dispersed, with trainees at varied institutions across the USA, which provided opportunities to collaborate across institutions. The interdisciplinary, culturally diverse makeup of the fellowship cohort lent itself to cooperative learning [31], broadening the fellowship focus from merely gaining knowledge from expert presenters and mentors to fostering a diverse community of learners sharing information. This finding was emphasized by a trainee: “The [different] areas of expertise of the trainees make for an exciting and collaborative experience.”

## Discussion

In our analysis of the ongoing evaluation activities of the A-TRAC fellowship, we report that the program effectively combined several important characteristics to support new scholars in TRS. The blended medium of webinars and annual in-person/virtual scientific meetings allowed for regular interaction among geographically dispersed trainees. Our experiential curriculum, based on adult-learning principles, engaged students to learn, practice, and incorporate the educational material into their research and lives. Intensive multilevel mentorship at the trainees’ home institutions, as well as across the broader field of TRS, helped to bond all project components to support trainees through critical stages of career and professional development. The focus on feedforward, including learning the value of giving and receiving helpful critiques, is vital to future professional development. Trainee diversity created a forum for multidisciplinary learning and teaching, providing opportunities for peer mentoring, as well as cross-disciplinary and cross-institutional collaborations. In examining the broader literature pertaining to multisite, multidisciplinary training and mentorship programs, we note that the A-TRAC program is unique in its focus on experiential learning, as well as the importance of feedback and feedforward for development and peer learning.

Through seminars, presentations, collaboration pairs, and informal discussion opportunities, trainees developed group cohesion, discussed sensitive topics in career and professional development, and engaged in opportunities for broader TRS networking. A potential weakness of online learning and training programs may involve the lack of connection and communication with trainers and mentors. In-person meetings were particularly effective for discussing complex and/or personal topics, such as work-life integration, resilience, mentorship challenges, and difficult interpersonal conversations, as well as for practicing presentation skills.

The focus on mentorship instilled a strong sense of accountability within the fellowship. Not only were trainees accountable for acquiring TRS knowledge and experiences, they also were expected to develop professionally by filing Docket comments with the FDA and submitting manuscripts and grant applications. Throughout the fellowship, participants reported on specific goals during one-on-one meetings and regularly communicated research progress during webinars to obtain feedback.

Although our analysis was based on program evaluation materials regularly collected from trainees throughout the A-TRAC fellowship, we identified other overarching components of the program that render it an appealing model for adoption in other fields: resource effectiveness, customizability, and scalability; needs-driven programming; and operations-level organization.

### Resource Effectiveness, Customizability, and Scalability

In our innovative model, A-TRAC trainees learned in a cohort structure, in which experienced research CEC mentors in the training core serve as secondary mentors in addition to the trainees’ institutional mentors. Our model was designed to capitalize on the resource-effectiveness of the blended instruction approach that allowed a set of diverse and geographically dispersed scholars to learn together. The costs of annual in-person meetings were offset by the negligible expense associated with weekly webinars, which trainees joined from any location with an internet connection. Since webinar presenters were located at research universities across the country, it would have been impractical and expensive to bring all these scholars on-site for weekly meetings in a traditional classroom format.

We envisioned that similar models could be employed to facilitate team science for other areas of research focus at both domestic and international levels. The A-TRAC model has served as a foundation for several other multisite and multi-institutional training programs within the AHA Strategically Focused Research Network (SFRN). The training program has been adapted by AHA SFRN Center grants including research training areas in Atrial Fibrillation [32], Cardiometabolic Health and Type 2 Diabetes, Health Technology, and Innovations and Disparities in Cardio-Oncology. Many adaptable concepts originally featured in A-TRAC – including the use of virtual multi-institutional mentored research and multilevel mentoring network – have been replicated in these individual training programs. In particular, such models are useful for connecting low-resource sites with higher-resource collaborators in order to facilitate shared learning among trainees. However, unique training considerations specific to A-TRAC are the regulatory implications of the research carried out by the trainees. Evidence generated by their research questions is used by the FDA to write policy on tobacco regulation. This regulatory framework and context may not be applicable to other training programs.

### Needs-Driven Program

The second central component of the A-TRAC fellowship’s success involved its focus on being driven by the needs of participants. We regularly captured program evaluation feedback to improve the fellowship. Trainees completed online pre-fellowship needs assessments, satisfaction surveys at the mid-point and end of each program year, and session-specific feedback forms after each webinar and in-person meeting. The systematic, consistent participant feedback helped to improve the program through filling gaps in TRS competencies and career development needs and providing cogent suggestions for future iterations of the fellowship.

### Operations-Level Organization

The third major component related to the success of the A-TRAC fellowship involved the operations-level organization. A program with this level of intensity and training was enabled by a Director of Training and Education. Previous and current A-TRAC Directors have possessed doctoral- or master’s-level training in related areas, including program evaluation, adult education, and public health and have maintained responsibility for managing and facilitating all components of training and program evaluation. The Director devoted time to meeting with each facilitator in-person or via videoconferencing at least once prior to sessions to assist with the development of curricula focused on principles of adult learning and spent time carefully considering changes to the curricula. The Director also was available to trainees for regular check-ins to maintain productivity and troubleshoot challenges.

### Limitations

Our analysis of the A-TRAC program has strengths and limitations. By analyzing ongoing program evaluations, we were able to observe areas of growth and development in both the program and the trainees – and to track how improvements in program implementation have been integrated over time.

However, we acknowledge substantive limitations. First, this is a study of program evaluation materials from one TRS program, limiting our ability to generalize to other programs or fields. Second, the evaluation relies on qualitative data analysis, and although we followed standard qualitative data analysis procedures, there exists a possibility for bias and oversight. Additionally, the number of program participants was relatively small, as our data were limited to the number of trainees enrolled in the first 7 years of the fellowship. However, the sample was relatively heterogeneous, with broad experience and histories to speak to the program’s overall function. The data sources also were from static time points and often had been designed to serve other purposes within the broader program evaluation.

## Conclusion

As technology continues to develop, connecting across distance becomes easier and more vital. For institutions considering new training and mentorship programs, creating web-focused interactive/experiential programming is promising and provides a scalable, efficient, and resource-effective educational method for fostering team science. Future work should consider both short- and long-term outcomes of the A-TRAC fellowship and similar programs, including career trajectories, publications, and successful grant applications.

## Supplementary material

For supplementary material accompanying this paper visit https://doi.org/10.1017/cts.2021.887. click here to view supplementary material

## References

[1] Ashley DL , Backinger CL , van Bemmel DM , Neveleff DJ. Tobacco regulatory science: research to inform regulatory action at the Food and Drug Administration’s Center for Tobacco Products. Nicotine & Tobacco Research 2014; 16(8): 1045–1049. 24638850 10.1093/ntr/ntu038

[2] FDA. Tobacco Science & Research, 2021. (https://www.fda.gov/tobacco-products/tobacco-science-research)

[3] Kleykamp BA , Gipson CD , Maynard OM , Treur JL , Oliver JA. Rethinking the career landscape for nicotine and tobacco trainees and early career professionals. Nicotine & Tobacco Research 2019; 21(2): 262–266. 29660013 10.1093/ntr/nty041 PMC6610160

[4] FDA. Achievements in Tobacco Regulation Over the Past Decade and Beyond, 2019. (https://www.fda.gov/news-events/fda-voices/achievements-tobacco-regulation-over-past-decade-and-beyond)

[5] National Institutes of Health. Tobacco Centers of Regulatory Science, 2021. (https://prevention.nih.gov/tobacco-regulatory-research/funded-research/funded-research-tobacco-centers-regulatory-science)

[6] Wipfli HL , Berman M , Hanson K , et al. Defining tobacco regulatory science competencies. Nicotine & Tobacco Research 2017; 19(2): 222–230. 27613917 10.1093/ntr/ntw178 PMC5234367

[7] Frenk J , Chen L , Bhutta ZA , et al. Health professionals for a new century: transforming education to strengthen health systems in an interdependent world. The Lancet 2010; 376(9756): 1923–1958. 10.1016/S0140-6736(10)61854-5 21112623

[8] Harden RM. Trends and the future of postgraduate medical education. Emergency Medicine Journal 2006; 23(10): 798–802. 16988312 10.1136/emj.2005.033738 PMC2579604

[9] Ruiz JG , Mintzer MJ , Leipzig RM. The impact of e-learning in medical education. Academic Medicine 2006; 81(3): 207–212. 16501260 10.1097/00001888-200603000-00002

[10] Cook DA , Triola MM. What is the role of e-learning? Looking past the hype. Medical Education 2014; 48(9): 930–937. 25113119 10.1111/medu.12484

[11] Rospigliosi P. Digital transformation of education: can an online university function fully? Interactive Learning Environments 2020; 28(8): 945–947.

[12] FDA. Comment on Proposed Regulations and Submit Petitions, 2019. (https://www.fda.gov/regulatory-information/dockets-management/comment-proposed-regulations-and-submit-petitions)

[13] Russo AR , Solis AC , Villanti AC , et al. Mentoring for success in tobacco regulatory science: a qualitative study. Tobacco Regulatory Science 2017; 3(3): 280–292. 28758143 10.18001/TRS.3.3.4 PMC5533280

[14] FDA. Tobacco Centers of Regulatory Science (TCORS), 2021. (https://www.fda.gov/tobacco-products/research/tobacco-centers-regulatory-science-tcors)

[15] FDA. Tobacco Centers of Regulatory Science (TCORS), 2018. (https://web.archive.org/web/20180725113926/https:/www.fda.gov/TobaccoProducts/PublicHealthScienceResearch/Research/ucm369005.htm)

[16] American Heart Association. Tobacco Centers of Regulatory Science (TCORS 2.0), 2020. (https://professional.heart.org/en/research-programs/a-trac)

[17] Akçayır G , Akçayır M. The flipped classroom: a review of its advantages and challenges. Computers & Education 2018; 126(6): 334–345.

[18] Kluger AN , Van Dijk D. Feedback, the various tasks of the doctor, and the feedforward alternative. Medical Education 2010; 44(12): 1166–1174. 21091758 10.1111/j.1365-2923.2010.03849.x

[19] Center for coordination of Analytics S, Enhancement, and Logistics (CASEL) in Tobacco Regulatory Science. TRS Knowledge Center, 2021. (https://trsknowledge.com)

[20] Di Frances CD , Childs E , Fetterman JL , et al. Implementing and evaluating a mentor training to improve support for early-career scholars in tobacco regulatory science. Nicotine & Tobacco Research 2020; 22(6): 1041–1045. 31095330 10.1093/ntr/ntz083 PMC7249929

[21] Strauss AL. Basics of Qualitative Research: Grounded Theory Procedures and Techniques. Newbury Park, CA: Sage Publications, 2013.

[22] University WG. Adult Learning Theories and Principles, 2020. (https://www.wgu.edu/blog/adult-learning-theories-principles2004.html)

[23] Nemiro JE . Creativity in virtual teams [Ph.D.]. Ann Arbor, The Claremont Graduate University; 1998.

[24] Goldsmith M. Try feedforward instead of feedback. The Journal for Quality and Participation 2003; 26(3): 38–40.

[25] Burt Ronald S. Structural holes and good ideas. American Journal of Sociology 2004; 110(2): 349–399.

[26] Bunderson JS , Sutcliffe KM. Comparing alternative conceptualizations of functional diversity in management teams: process and performance effects. Academy of Management Journal 2002; 45(5): 875–893.

[27] Piaget J. The Child’s Conception of the World. New York: Harcourt, Brace and Company, 1929.

[28] Piaget J. The Moral Judgment of the Child. 1st Free Press paperback edition. New York: Free Press, 1965.

[29] Vygotski&ibreve; LS. Mind in Society: The Development of Higher Psychological Processes. Cambridge: Harvard University Press, 1978.

[30] Knowles MS. The Adult Learner: The Definitive Classic in Adult Education and Human Resource Development. 8th ed. New York: Routledge, 2015.

[31] Berndt TJ , Ladd GW. Peer Relationships in Child Development. New York, NY: Wiley, 1989.

[32] Ajayi TB , Remein CD , Stafford RS , et al. Cross-center virtual education fellowship program for early-career researchers in atrial fibrillation. Circulation: Arrhythmia and Electrophysiology 2020; 13(11): e008552. 33031707 10.1161/CIRCEP.120.008552 PMC7674267

