# Supplementary material for: Fostering Tobacco Regulatory Team Science through a multisite, virtual fellowship program for early-career researchers
Source: J Clin Transl Sci. 2022 Jan 4;6(1):e14. doi: 10.1017/cts.2021.887 (PMC8826010; doi:10.1017/cts.2021.887)
Supplement: Supplementary file 1 [file S2059866121008876sup001.docx]

**Supplement 1. Needs assessment questions in entrance survey for trainees**

| **Entrance survey questions** | |
| --- | --- |
| Q.1 Demographic: Name, Degree, Institution and Project Title | Q.4 What professional development-related topics would you most like to see included in the curriculum? |
| Q.2 What specific issues around Tobacco Regulatory Science (TRS) are you most interested in? | Q.5 What do you see as your most important areas for development as you start this fellowship? |
| Q.3 What scientific topics would you most like to see included in the curriculum? | Q.6 What would you define as successful outcomes for the fellowship? |

***Questions given on the entrance survey completed by all trainees**

**Supplement 2.** **Program Components of the A-TRAC Fellowship**


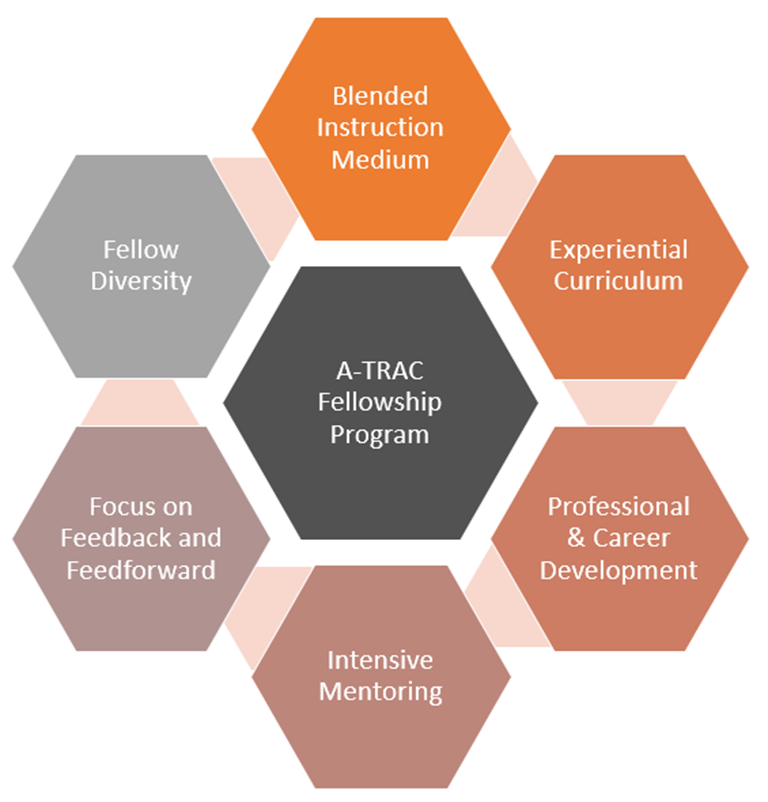


**Supplement 3. A-TRAC Fellowship Program Facilitator’s guide**

| **Session Title** |  |
| --- | --- |
| **Session Facilitator** |  |
| **Date** |  |
| **Learning Objectives** |  |
| **Related Competencies** |  |
| **Reading/**  **resources** |  |
| **Poem/quote/art/song*** |  |
| **Participant Preparation** |  |
| **Participant Commitment/**  **SMART Goal** |  |
| **Materials needed** |  |

| **Topic** | **Content** | **Activity/Pedagogy** | **Time** |
| --- | --- | --- | --- |
|  |  |  |  |
|  |  |  |  |
|  |  |  |  |
|  |  |  |  |
|  |  |  |  |
|  |  |  |  |
|  |  |  |  |

* Optional
